# HIV/AIDS Knowledge, Attitudes, and Behaviors of Construction Workers in China

**Published:** 2008-09

**Authors:** Bo Qu, Haiqiang Guo, Gao Sun, Tianming Zuo, Yang Zhang, Brandon Y. Li

**Affiliations:** 1 *School of public health, China Medical University, Shenyang, China;*; 2 *Center for medical education, China Medical University, Shenyang, China;*; 3 *University of Southern California, USA*

**Keywords:** HIV/AIDS, knowledge, attitude, behavior, China

## Abstract

The objective of the study was to describe HIV/AIDS knowledge, attitudes, risk behaviors, and sources of information among construction workers in China. A cross-sectional survey of 458 construction workers was conducted among 4 construction sites in Shenyang city in 2006. All 458 participants were individually interviewed in a private setting by a trained team of medical researchers using a structured questionnaire, which included questions on general personal information and the knowledge, attitudes, practice questions and the favorable mode of health education. A total of 428 valid questionnaires were collected. Data entry and statistical analyses were performed using the SPSS 13.0. Our results indicated that the majority of construction workers in China are sexually active youths and adults with limited education and poor knowledge of HIV/AIDS. The proportions of correct answers to questions about HIV/AIDS ranged from 4.9% to 70.7%. The score was significantly different by education level (χ^2^=47.51, *p*<0.01), and marrital status (χ^2^=16.48, *p*<0.01). More than 60% of the construction workers had a negative attitude toward HIV/AIDS-infected individuals. The source of workers’ knowledge toward HIV/AIDS mainly came from TV (35.8%), newspaper (14.3%), family and friend (13.1%) and others (28.2%). Chinese migrant workers in general lack knowledge about HIV/AIDS. Our study suggests prevention programs should be encouraged and these may have the potential role to limit the emergence of China’s HIV/AIDS epidemic.

## INTRODUCTION

China’s HIV/AIDS epidemic has gone through two stages (‘entry’, 1985-88, ‘spreading’ 1989-94). It is now in the third stage - ‘expansion’. The UNAIDS estimates that 10 million Chinese may be infected with HIV/AIDS by 2010 (1). HIV infection has begun to spread beyond the initial transmission pockets of injection drug users and blood transfusions (2-4). There is new evidence suggesting that a large majority of China’s migrants have originated from regions with more severe HIV/AIDS epidemics and their risk behaviors will change after migration.

The common characteristics of the “floating” population of migrant workers include more males, younger age group, lower education level, and more short-term and heavier labor (5-8). According to the Ministry for Public Security, there were approximately 40 million registered migrants in 1998, compared with some unofficial estimates of 80 million (9). As they generally have limited access to television or radio programs, health education has not reached them effectively. Public health programs must work out ways to include this part of the population.

Construction workers comprise the majority of the floating population in China, but there has been little investigation into HIV/AIDS and related issues among these workers. Many studies on HIV/AIDS-related knowledge, attitudes, practices, and intervention of HIV/AIDS have been carried out in the general population, with particular interest among subpopulations such as prostitutes, drug users, homosexual men, and so on. In this study, we conducted a population-based survey among construction workers in Shenyang, China to investigate HIV/AIDS related knowledge, attitude, and behavior among this population sub-group.

## METHODS

### Design

A cross-sectional design was employed. A pilot-investigation of 50 randomly sampled construction workers was carried out one month before the formal investigation of the whole group. The purpose of the pilot-investigation was to select the most representative items from the questionnaire to ensure the structure validity and increase the reliability of the investigation.

### Data collection methods

We used a self-administered HIV/AIDS questionnaire written in Chinese, the content of which was initially drafted following by a previous study conducted in China (Chen *et al*. 2004; Huang *et al*. 2005). Moreover, additional content was added to the questionnaire based on other relevant previous studies.

All 458 participants were individually interviewed in a private setting by a team of trained medical researchers using a self-developed questionnaire, which included questions on general personal information and the knowledge, attitude, practice questions, and the favorable mode of health education. In total, 428 valid questionnaires were collected.

### Ethical considerations

The study was an anonymous survey. A self-administered questionnaire comprised the two instruments. Return of a completed questionnaire indicated consent to participate in the study. The questionnaire did not contain any identifying information. The participation in the survey was totally voluntary. Participants had the option of declining to answer specific questions or leaving the entire questionnaire blank if they wished not to participate.

### Data analysis

One member of the research team assembled hard copies of the questionnaires and oversaw data cleaning, entry and analysis. Data entry and analysis were conducted using SPSS 13.0 (citation here). Percentage distributions of knowledge, attitude, and practice of HIV/AIDS were tabulated and χ^2^ test was used to analyze it.

## RESULTS

### Study population

Shenyang City is the capital of Liaoning Province, located in the north of China. It has a population of 7.2 million in 2006. We selected randomly 4 construction sites for the investigation from Shenyang City. In this study, construction workers were individually interviewed in a private setting by a team of trained medical researchers using a self-developed questionnaire, which included general personal information and the knowledge, attitude, practice questions and the favorable mode of health education. Out of all 458 construction workers, 428 completed their questionnaires, representing an overall response rate of 93.4%.

All 428 construction workers between the age of 18 and 52 years old in those sections were invited to participate in the survey. All participants are male. This studied population is 72.4% married, 26.1% single, and 15% divorced. Their educational level are varied: illiteracy (10.9%), elementary school (28.1%), junior high school (54.8%), senior high school and upwards (6.2%). These workers mainly come from Sichuan province (26.2%), Liaoning province (14.1%), Henan province (14.5%), Hubei province (11.1%), and other provinces (34.1%).

### The analysis of the reliability and validity

The pilot-investigation of the 50 construction workers had a Cronbach’s alpha coefficient of 0.78. An exploratory factor analysis (EFA) was first applied to ensure the structural validity. The analysis was performed using principal axis factoring with varimax rotation on the correlations of the observed variables. The Bartlett’s test of sphericity was found to be significant (*p*<0.01), while the Kaiser–Meyer–Olkin measure of sampling adequacy was 0.72, justifying the application of the factor-analytic procedure.

One month later, the formal investigation was carried out and the Cronbach’s alpha coefficient of this study was 0.86. The factor analysis demonstrates that the items listed in the quesitonnaire accounting for 88.1% of the variance.

### Knowledge of HIV/AIDS

Table 1 shows that 70.1% and 70.3% of participants answered correctly to the two respective questions about the awareness of HIV transmission routes: “HIV can be transmitted by sharing needles for drug use with someone who has HIV?” and “A pregnant woman with HIV can give the virus to her baby”. However, the proportions of subjects who know the non-transmission routes were low, varying between 4.9% and 35.5%. It is promising for the future of HIV/AIDS that 70.7% subjects agreed AIDS can be prevented. A total score was calculated by adding the score of each question, which account for 0 to wrong answer, 1 to don’t know and 2 to correct answer. The total knowledge score was significantly different by education level (*t*=25.32, *p*<0.01).

**Table 1:** The survey result of HIV/AIDS knowledge (%)

HIV/ AIDS Knowledge item	Agree	Disagree	Don’t know

AIDS is a sexually transmitted disease.	206 (48.1%)	67 (15.6%)	155 (36.2%)
There is no cure for AIDS.	224 (52.3%)	107 (25.0%)	97 (22.7%)
AIDS can be prevented.	303 (70.7%)	36 (8.4%)	89 (20.7%)
A pregnant woman with HIV can give the virus to her baby.	301 (70.3%)	42 (9.8%)	85 (19.9%)
HIV can be transmitted by sharing needles for drug use with someone who has HIV.	300 (70.1%)	25 (5.8%)	103 (24.1%)
Can you get HIV from the following sources?			
Eating in a restaurant where the cook has HIV?	175 (40.9%)	152 (35.5%)	101 (23.6%)
Mosquitoe or other insect?	284 (66.4%)	21 (4.9%)	123 (28.7%)
Being coughed or sneezed on by someone who has HIV?	186 (43.5%)	119 (27.8%)	123 (28.7%)
Using public toilets?	164 (38.3%)	100 (23.3%)	164 (38.3%)
Swimming with someone who has HIV?	178 (41.6%)	87 (20.3%)	163 (38.1%)

### Attitudes toward HIV/AIDS

The results show that people had prejudiced attitudes towardsAIDS patients and can’t accept living with AIDS patients. 82% of construction workers thought that people with HIVshould be isolated; 62.6% of subjects were not willing to have meals with people who have HIV; but fortunately, 87.1% of respondents considered that people with HIV should be helped more; and 73.6% of subjects were willing to take care of families of patients with HIV.

### HIV/AIDS-related risk behaviors

Table 2 shows that some of the interviewed subjects were at risk of HIV infection. Among the participants, 63.0% thought that only easily infectious people with HIV would be prevented, 19.9% did not think that extramarital sex could result in AIDS, and 32.7% disagreed that drug use would transmitted HIV.

**Table 2:** Builders’ Attitudes toward HIV/AIDS patients and high-risk behaviors

Attitudes and behavior of HIV/AIDS item	Strongly agree	agree	Disagree

People with HIV/AIDS should be insulated	295 (68.9%)	56(13.1%)	77 (18.0%)
Extramarital sex would lead to HIV infection or AIDS	324 (75.7%)	19 (4.4%)	85 (19.9%)
Drug users would infect HIV/AIDS	232 (54.2%)	56 (13.1%)	140 (32.7%)
Only impressionable people with HIV/AIDS would be prevented	261 (61.0%)	25 (5.8%)	142 (33.2%)
People with HIV/AIDS should be offer more help	343 (80.1%)	30 (7.0%)	55 (12.9%)
I am willing to have meals with HIV carriers/AIDS patients	86 (20.0%)	74 (17.3%)	268 (62.6%)
I am willing to take care of family members with HIV/AIDS	259 (60.6%)	55 (12.9%)	114 (26.6%)

### Source and requirement of information about HIV/AIDS

Our study shows that the source of information about AIDS includes TV (35.8%), newspaper (14.3%), family and friend (13.1%), and radio (4.2%). A total of 82.2% of construction workers wanted to acquire more HIV/AIDS knowledge. The favorite modes of learning are “Doctor’s lectures or consultation toward AIDS” (28.3%), “providing booklet” (16.6%), “looking on CD” (14.7%).

## DISCUSSION

The construction workers we studied are mostly sexually-active youth and adults from rural areas with low education level and low income. Our study shows the HIV/AIDS knowledge among these workers is related to the level of education. The lower the education level, the less knowledge the construction workers have about HIV/AIDS. It will be necessary to improve the education level of rural people. Because of these workers’ low-income, an orderly medical physical examination is not feasible. In addition to discrimination against HIV/AIDS patients, they weren’t expected to be HIV tested, which increases the possibility of unknown spread of HIV.

Our investigation revealed that the majority of construction workers in Shenyang were migrants from Sichuan province (26.2%) and Henan province (14.5%). This segment of construction workers may play an important role in spreading the disease. We must pay attention to these people’s HIV testing and education to decrease the lack of awareness regarding HIV prevention. Still, the main transmission route of HIV is through sexual contact, rather than blood transfusion and drug-use.

Our study shows that construction workers have little knowledge about HIV risk behaviors, which makes them at greater risk of contracting HIV. In promoting and disseminating HIV/AIDS knowledge, we can select TV, newspaper, and family-friend interchanges. Most of construction workers (82.2%) wanted to know more about HIV/AIDS and 60.2% wanted to take part in a free course of lectures. So we can make use of these ways to propagate the HIV/AIDS knowledge.

There are some limitations in this research. The study was limited to four building sites. Another limitation is that the study doesn’t show any information about any risky sexual behaviors, therefore can not determine the risk levels for this group. Because of the limited conditions, we can’t investigate the construction workers’ HIV infection rate, which is estimated to be higher than the rate among other population groups. Finally, only 428 construction workers were included in our study; future studies with bigger sample size are needed.

Based on our findings, we suggest: (a) we must pay more attention to construction workers in China in order to prevent the HIV/AIDS epidemic from spreading quickly; (b) education programs are in urgent need for this group of people and such programs should aim at both equipping these individuals with necessary knowledge about HIV/AIDS and correcting misunderstandings; (c) HIV/AIDS interventions should incorporate programs that can help construction workers improve their income level because they have a strong desire in this regard; (d) a free course of HIV/AIDS lectures should be regarded as a useful tool in planning interventions.

## CONCLUSION

We found that construction workers mostly came from rural areas, had low education levels, were sexually active, and had low incomes. They have little knowledge about HIV/AIDS and related risk behaviors. China should implement HIVAIDS prevention programs for this population group.
